# Supplementary material for: Using the drug-protein interactome to identify anti-ageing compounds for humans
Source: PLoS Comput Biol. 2019 Jan 9;15(1):e1006639. doi: 10.1371/journal.pcbi.1006639 (PMC6342327; doi:10.1371/journal.pcbi.1006639)
Supplement: S1 Text — Research bias and influence of the cutoff selection. (DOCX) [file pcbi.1006639.s002.docx]

**Drugs significantly enriched for ageing-related targets**

The list of pro-longevity compounds identified using “gene” information included **resveratrol**, which was initially reported to extend the lifespan of yeast [1]. Similar findings followed in worms and flies [2], which were later confirmed [3] and challenged [4]. In mice, resveratrol recovered the longevity of animals on a lifespan-shortening, high-calorie diet, but not of mice on a normal diet, although these mice showed some delayed signs of ageing [5]. Human clinical trials have reported that resveratrol influences biomarkers of Alzheimer’s disease and cardiovascular disorders beneficially, but inconclusive and occasionally detrimental effects have been observed for certain cancers [6]. Another prioritised compound was **simvastatin**, which increases lifespan and improves cardiac function of *Drosophila* [7] but not in mice [8]. Similarly, combination therapy of simvastatin and ramipril significantly increased lifespan by 9 % in long-lived B6C3F1 mice, but no effect was detected for simvastatin or ramipril alone [9]. Interestingly, predictions based on 5.8 years clinical trial in patients with coronary heath disease taking simvastatin indicates that lifetime treatment could increase survival up to 9.9 years [10]. Another widely studied compound present in our list is **epigallocatechin gallate,** which increases the lifespan of worms [11] and flies [12]. In rats, it extends lifespan by 13%, by reducing inflammation and oxidative stress in liver and kidney tissues through inhibition of NF-kB and activation of FOXO3A and SIRT1 [13]. Some findings in humans have been inconclusive [14], possibly because of the low and erratic bioavailability of this compound, while others have been positive. For example, a clinical trial for prostate cancer chemoprevention with a daily dose of 200 mg green tea catechin (51% epigallocatechin gallate) reduced by 90% the rate of high-grade prostate intraepithelial, neoplasia-positive men developing prostate cancer [15]. A study in healthy humans recently showed that food inhibits the bioavailability of epigallocatechin gallate [16], so further testing of this compound in new clinical trials or experiments in animal models is needed. In contrast, other compounds such as **sirolimus** have shown similar anti-ageing properties in dozens of independent studies [17]. However, this drug has higher bioavailability, especially the encapsulated version, which makes it a good candidate for further studies in humans. Another pro-longevity compound in the top-ranked list is **genistein**. This compound induces a concentration-dependent lifespan extension in nematodes and improves their thermo-tolerance, stress resistance and body movement [18]. These benefits were associated with an elevated superoxide dismutase and catalase activity. In contrast, a study published in 2011 reported that genistein shortens the lifespan of *D. melanogaster* in doses ranging from 1uM to 10uM [19]. In a type 2 diabetes rat model (Zucker diabetic fatty), genistein was demonstrated to prevent cardiac dysfunction and pathological alterations by reducing oxidative stress and improving glucose and insulin homeostasis [20]. Finally, **celecoxib** increases the lifespan of *C. elegans* up to 20% by inhibiting the 3′-phosphoinositide-dependent kinase1 (PDK-1) in the insulin/IGF signalling pathway [21]. The inhibition of PDK-1 by celecoxib also has been shown to induce apoptosis in human colon cancer cells [22]. Likewise, flies fed with a derivative of celecoxib lacking cyclooxygenase-2 inhibitory function (2,5-Dimethyl-celecoxib) live longer and have improved stress tolerance, physical activity and intestinal integrity [23].

**Interaction-based similarity analysis**

Because drugs with shared known targets may have a similar mechanism of action and consequently influence ageing in the same direction, we evaluated the similarities between the targets of drugs were calculated using a signed version of the Jaccard index previously employed to compare drugs effects at the transcriptomic level [24]. Genes targeted by only one drug were removed to minimise the overestimation of differences between the drugs. The standard Jaccard index corresponds to the number of proteins targeted by both drugs divided by the total number of targets of both drugs. However, the signed version is calculated by adding the indexes for the targets in which both drugs are acting in the same direction (e.g. the comparison is made between proteins activated by each drug) minus the index for the targets where one drug is acting opposite to the other (e.g. proteins activated by one drug are compared with proteins inhibited by the other drug). Because each index ranges from 0 to 1 the subtraction is divided by 2, resulting in an index ranging from -1 to 1, where values closer to -1 represents drugs acting on similar targets but in opposite directions while a value of 1 is assigned for two drugs with the same interaction pattern (Fig 1A). Drugs were clustered hierarchically based on their similarities using Ward’s method [25].

**Fig 1. Interaction-similarities between the top-ranked compounds.** A) Scheme of the interactions similarity between two drugs. Interactions with both drugs in the same direction are shown as blue lines while opposite interactions are displayed in red. B) A positive value (blue) represents a similar interaction pattern, a negative value (red) opposite interaction pattern. The values from the diagonal were removed for better display of the indexes between different compounds. Annotations were added in the upper part of the heatmap to indicate if the drugs were in the DrugAge database and their current development status. Row and columns were clustered by similarity values, and the calculated hierarchical tree is shown at the top.

One of the two main branches of the tree is composed exclusively of pro-ageing compounds, namely doxorubicin, cisplatin and hydrogen peroxide, while all pro-longevity drugs are in the other branch (Fig 1B). Four pro-longevity drugs (genistein, resveratrol, epigallocatechin gallate, sirolimus) cluster relatively closely. cAMP shows a similar interaction pattern to resveratrol and genistein, while selenium and dorsomorphin display a different interaction pattern from the other drugs. Bexarotene showed high similarity with celecoxib and GW-501516. As expected, the kinase inhibitors sunitinib, regorafenib and sorafenib cluster together. Tanespimycin has a similar interaction pattern to simvastatin.

Interestingly, GW-501516 had an opposite interaction pattern to the pro-ageing compounds. Something similar was observed for selenium, which was dissimilar to sorafenib, simvastatin, celecoxib and hydrogen peroxide. Inspection of the pairwise similarity values revealed that the highest similarity was found for sorafenib and regorafenib followed by GW-501516 and bexarotene. The pro-longevity drugs sirolimus and epigallocatechin gallate also showed a similar interaction pattern as well as the neoplastic agents doxorubicin and cisplatin. Notably, the similarity analysis agreed with the literature, being able to cluster the pro-ageing drugs and separate them from the remaining drugs. Likewise, the similar interaction pattern of 4 drugs with pro-longevity effects suggest the existence of shared anti-ageing mechanism between these drugs.

**Measuring the impact of research bias**

**Table A. Evaluation of the research bias for the top-ranked compounds on each list.** The first column represents the type of data used as the comparator between ageing and drug targets. The p-value on the second column corresponds to the higher p-value obtained for the 20 compounds on the top of each list, and it represents how many of the 1000 permutations showed a higher rank than in the analysis.

| Data source | p-value |
| --- | --- |
| Genes | 0.012 |
| PPI | 0.012 |
| GO:BP | 0.007 |
| GO:CC | 0.010 |
| GO:MF | 0.017 |
| KEGG | 0.005 |
| Reactome | 0.010 |

**Enrichment for pro- and anti-longevity drugs using different biological levels**

**Fig 2. Area under the enrichment curves calculated using different cutoff values.** The solid lines represent the enrichment for pro-longevity drugs and the dashed lines for anti-longevity drugs. We analysed 3 cutoffs for the confidence score (600, 700, 800) and the adjusted p-value (0.01, 0.05, 0.1). The colour of the lines and dot represent the use of different types of data as comparator.

**Table B. Evaluation of the statistical significance of the enrichment for pro-longevity compounds.** The first column represents the type of data used as the comparator between ageing and drug targets. The second columns show the p-value, which represents how many of the 1000 permutations used to evaluate the research bias, showed an AUC equal or higher than the analysis.

| Data source | p-value |
| --- | --- |
| Genes | <0.001 |
| PPI | 0.001 |
| GO:BP | 0.027 |
| GO:CC | 0.005 |
| GO:MF | <0.001 |
| KEGG | 0.001 |
| Reactome | <0.001 |

**References**

1. Howitz KT, Bitterman KJ, Cohen HY, Lamming DW, Lavu S, Wood JG, et al. Small molecule activators of sirtuins extend Saccharomyces cerevisiae lifespan. Nature. 2003;425: 191–196. doi:10.1038/nature01960

2. Wood JG, Regina B, Lavu S, Hewitz K, Helfand SL, Tatar M, et al. Sirtuin activators mimic caloric restriction and delay ageing in metazoans. Nature. Nature Publishing Group; 2004;430: 686–689. doi:10.1038/nature02789

3. Bauer JH, Goupil S, Garber GB, Helfand SL. An accelerated assay for the identification of lifespan-extending interventions in Drosophila melanogaster. Proc Natl Acad Sci. 2004;101: 12980–12985. doi:10.1073/pnas.0403493101

4. Farghali H, Kutinová Canová N, Lekić N. Resveratrol and related compounds as antioxidants with an allosteric mechanism of action in epigenetic drug targets. Physiol Res. 2013;62: 1–13. doi:10.1016/j.mad.2007.07.007

5. Pearson KJ, Baur JA, Lewis KN, Peshkin L, Price NL, Labinskyy N, et al. Resveratrol Delays Age-Related Deterioration and Mimics Transcriptional Aspects of Dietary Restriction without Extending Life Span. Cell Metab. 2008;8: 157–168. doi:10.1016/j.cmet.2008.06.011

6. Berman AY, Motechin RA, Wiesenfeld MY, Holz MK. The therapeutic potential of resveratrol: a review of clinical trials. npj Precis Oncol. 2017;1: 35. doi:10.1038/s41698-017-0038-6

7. Spindler SR, Li R, Dhahbi JM, Yamakawa A, Mote P, Bodmer R, et al. Statin treatment increases lifespan and improves cardiac health in Drosophila by decreasing specific protein prenylation. PLoS One. United States; 2012;7: e39581. doi:10.1371/journal.pone.0039581

8. Miller RA, Harrison DE, Astle CM, Baur JA, Boyd AR, De Cabo R, et al. Rapamycin, but not resveratrol or simvastatin, extends life span of genetically heterogeneous mice. Journals Gerontol - Ser A Biol Sci Med Sci. 2011;66 A: 191–201. doi:10.1093/gerona/glq178

9. Spindler SR, Mote PL, Flegal JM. Combined statin and angiotensin-converting enzyme (ACE) inhibitor treatment increases the lifespan of long-lived F1 male mice. Age (Omaha). 2016;38: 379–391. doi:10.1007/s11357-016-9948-4

10. Chang S-C, Wang M, Beckerman JG, Sanz M, Gluckman TJ, Grunkemeier GL. A method to estimate the mean lifetime survival increase of statin therapy. Future Cardiol. England; 2016;12: 539–544. doi:10.2217/fca-2016-0016

11. Abbas S, Wink M. Epigallocatechin gallate from green tea (Camellia sinensis) increases lifespan and stress resistance in Caenorhabditis elegans. Planta Med. 2009;75: 216–221. doi:10.1055/s-0028-1088378

12. Wagner AE, Piegholdt S, Rabe D, Baenas N, Schloesser A, Eggersdorfer M, et al. Epigallocatechin gallate affects glucose metabolism and increases fitness and lifespan in &lt;i&gt;Drosophila melanogaster&lt;/i&gt; Oncotarget. 2015;6: 30568–30578. doi:10.18632/oncotarget.5215

13. Niu Y, Na L, Feng R, Gong L, Zhao Y, Li Q, et al. The phytochemical, EGCG, extends lifespan by reducing liver and kidney function damage and improving age-associated inflammation and oxidative stress in healthy rats. Aging Cell. 2013;12: 1041–1049. doi:10.1111/acel.12133

14. Mähler A, Mandel S, Lorenz M, Ruegg U, Wanker EE, Boschmann M, et al. Epigallocatechin-3-gallate: a useful, effective and safe clinical approach for targeted prevention and individualised treatment of neurological diseases? EPMA J. Springer; 2013;4: 5. doi:10.1186/1878-5085-4-5

15. Bettuzzi S, Brausi M, Rizzi F, Castagnetti G, Peracchia G, Corti A. Chemoprevention of human prostate cancer by oral administration of green tea catechins in volunteers with high-grade prostate intraepithelial neoplasia: A preliminary report from a one-year proof-of-principle study. Cancer Res. 2006;66: 1234–1240. doi:10.1158/0008-5472.CAN-05-1145

16. Naumovski N, Blades B, Roach P. Food Inhibits the Oral Bioavailability of the Major Green Tea Antioxidant Epigallocatechin Gallate in Humans. Antioxidants. 2015;4: 373–393. doi:10.3390/antiox4020373

17. Johnson SC, Kaeberlein M. Rapamycin in aging and disease: maximizing efficacy while minimizing side effects. Oncotarget. Impact Journals, LLC; 2016;7: 44876–44878. doi:10.18632/oncotarget.10381

18. Lee EB, Ahn D, Kim BJ, Lee SY, Seo HW, Cha YS, et al. Genistein from vigna angularis extends lifespan in caenorhabditis elegans. Biomol Ther. 2015;23: 77–83. doi:10.4062/biomolther.2014.075

19. Altun D, Uysal H, AşkIn H, Ayar A. Determination of the effects of genistein on the longevity of Drosophila melanogaster Meigen (Diptera; Drosophilidae). Bull Environ Contam Toxicol. United States; 2011;86: 120–123. doi:10.1007/s00128-010-0159-x

20. Tian HS, Zhou GQ, Zhu ZY. Evaluation of cardioprotective effects of genistein against diabetes-induced cardiac dysfunction in rats. Trop J Pharm Res. 2015;14: 2015–2022. doi:10.4314/tjpr.v14i11.10

21. Ching TT, Chiang WC, Chen CS, Hsu AL. Celecoxib extends C. elegans lifespan via inhibition of insulin-like signaling but not cyclooxygenase-2 activity. Aging Cell. 2011;10: 506–519. doi:10.1111/j.1474-9726.2011.00688.x

22. Arico S, Pattingre S, Bauvy C, Gane P, Barbat A, Codogno P, et al. Celecoxib induces apoptosis by inhibiting 3-phosphoinositide-dependent protein kinase-1 activity in the human colon cancer HT-29 cell line. J Biol Chem. 2002;277: 27613–27621. doi:10.1074/jbc.M201119200

23. Wu Q, Lian T, Fan X, Song C, Gaur U, Mao X, et al. 2,5-Dimethyl-Celecoxib Extends Drosophila Life Span via a Mechanism That Requires Insulin and Target of Rapamycin Signaling. Journals Gerontol - Ser A Biol Sci Med Sci. 2017;72: 1334–1341. doi:10.1093/gerona/glw244

24. Wang Z, Monteiro CD, Jagodnik KM, Fernandez NF, Gundersen GW, Rouillard AD, et al. Extraction and analysis of signatures from the Gene Expression Omnibus by the crowd. Nat Commun. 2016;7: 12846. doi:10.1038/ncomms12846

25. Ward JH. Hierarchical Grouping to Optimize an Objective Function. J Am Stat Assoc. Taylor & Francis, Ltd.American Statistical Association; 1963;58: 236–244. doi:10.1080/01621459.1963.10500845
