# Supplementary figures and images for: Using the drug-protein interactome to identify anti-ageing compounds for humans
Source: PLoS Comput Biol. 2019 Jan 9;15(1):e1006639. doi: 10.1371/journal.pcbi.1006639 (PMC6342327; doi:10.1371/journal.pcbi.1006639)

**A**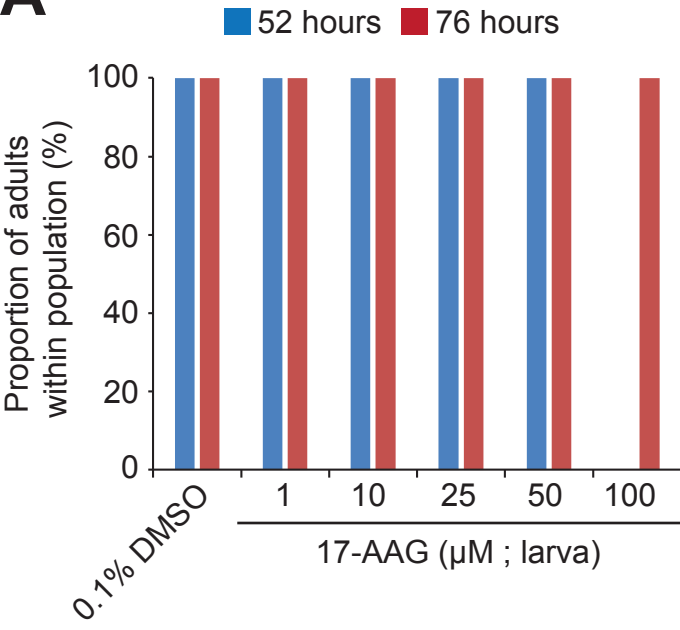**B**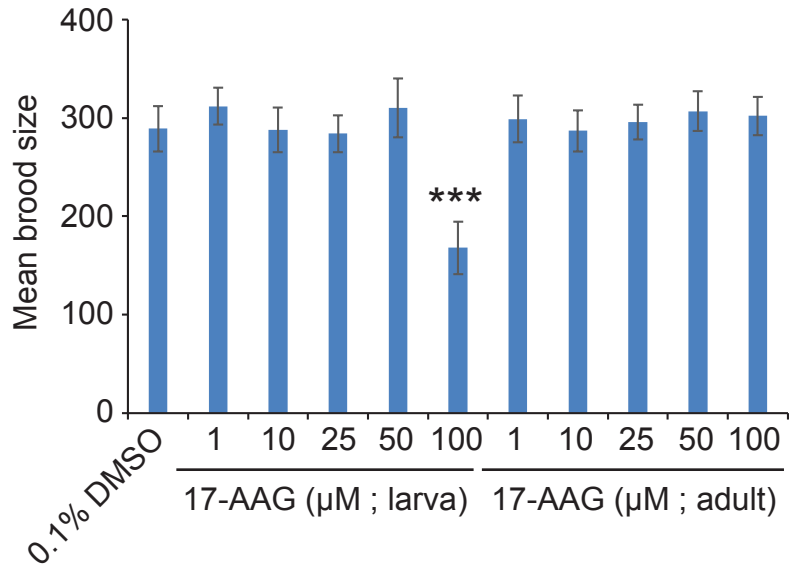

Supplement: S1 Fig — A) Proportion of the population that had reached adulthood 52 and 76 hours post seeding of L1 worms to plates containing 0.1% DMSO or increasing concentrations of 17-AAG. B) Total number of progeny produced by worms exposed to 0.1% DMSO or increasing concentrations of 17-AAG from the first larval stage (L1) onward or from the first day of adulthood. The number of progeny produced by individual worms on days 1 to 5 of adulthood was counted and combined. 10 worms were scored per treatment group and values plotted are the mean. Error bars denote standard deviation. Statistical significance was calculated by one-way ANOVA with Tukey pairwise comparison of groups. *** = p < 0.001. (PDF) [file pcbi.1006639.s003.pdf]
